# Satisfaction with Social Support Received from Social Relationships in Cases of Chronic Pain: The Influence of Personal Network Characteristics in Terms of Structure, Composition and Functional Content

**DOI:** 10.3390/ijerph17082706

**Published:** 2020-04-15

**Authors:** Rosario Fernández-Peña, José Luis Molina, Oliver Valero

**Affiliations:** 1 Department of Nursing, University of Cantabria (Spain), SALBIS Research Group, Nursing Research Group IDIVAL, 39008 Santander, Spain; 2 Department of Social and Cultural Anthropology, GRAFO, Universitat Autònoma de Barcelona, 08193 Barcelona, Spain; joseluis.molina@uab.cat; 3 Servei d’Estadística Aplicada, Universitat Autònoma de Barcelona, 08193 Barcelona, Spain; oliver.valero@uab.cat

**Keywords:** social support, patient satisfaction, chronic disease, chronic pain

## Abstract

The worldwide burden of chronic illnesses, constitutes a major public health concern and a serious challenge for health systems. In addition to the strategies of self-management support developed by nursing and health organizations, an individual’s personal network represents a major resource of social support in the long-term. Adopting a cross-sectional design based on personal network analysis methods, the main aim of this study is to explore the relationship between satisfaction with the social support received by individuals suffering chronic pain and the structure, composition, and functional content in social support of their personal networks. We collected personal and support network data from 30 people with chronic pain (20 person’s contacts (alters) for each individual (ego), 600 relationships in total). Additionally, we examined the level of satisfaction with social support in each of the 600 relationships. Bivariate and multivariate tests were performed to analyze the satisfaction with the social support received. Using cluster analysis, we established a typology of the 600 relationships under study. Results showed that higher satisfaction was associated with a balance between degree centrality and betweenness (i.e., measures of network cohesion and network modularity, respectively). Finally, new lines of research are proposed in order to broaden our understanding of this subject.

## 1. Introduction

Chronic illness constitutes a critical public health challenge that affects both social and economic development worldwide. Chronic illness also represents a major challenge for health services. Data from the WHO reveals that noncommunicable diseases (NCDs) cause 41 million deaths annually, equivalent to 71% of all deaths globally [1].

The steady increase in the prevalence of chronic illnesses is due, in part, to the progressive aging of the population, together with an increased life expectancy. Thus, people with several illnesses or chronic conditions live longer than in the past [2]. Faced with this scenario, health services are undergoing a reorganizational shift from a model centered on illness and treatment, to one centered on the individual and their particular chronic conditions. Both institutional and personal environments are also very important to chronic illness patients, comprising sources of support outside the more formal health and/or social systems.

A key element of the models developed to face chronicity is self-management. This concept emphasizes the patient’s role in managing health and has been defined as an ‘individual’s ability to manage the symptoms, treatment, physical, and psychosocial consequences and lifestyle changes inherent in living with a chronic condition’ [3]. This is a changing construct, which reflects the different types of support needed throughout the progression of the illness, mediated by such factors as the stage of the illness, its stability over time, the symptoms, and the physical limitations, among others [4]. However, as proposed by Morris et al. [5], the notion of self-management is questionable, as an individual construct, namely because many practices concerning the management of illnesses involve the support and/or negotiation of roles within the framework of relationships. Therefore, in essence, chronic illnesses are not restricted to the individual, rather they are ‘embedded in family, community, and societal conditions that shape and influence—and may constrain—the choices people make, or can make’ [6]. Based on this change in paradigm, the Innovate Care for Chronic Conditions (ICCC) of the WHO, propose a framework for health systems to improve the management of chronic conditions. This framework proposes the following micro, meso, and macro levels of health care systems: The level of interaction with the patient (micro), the organization of healthcare at the community level (meso), and the level of politics (macro). Each level dynamically interacts and influences the other two over time, via feedback loops [7]. According to the ICCC framework, optimal outcomes are achieved when a partnership exists between health care teams, community partners, and patients and their families [8], i.e., when individual self-management is embedded in the social systems of support that make it possible. Furthermore, certain aspects of the community are highly relevant, such as associations for patients and their families, making caregivers increasingly influential [9].

Along these lines, different dedicated programs and interventions have been developed by health organizations to improve self-management, including assessments based on the perspectives of both patients and health professionals [10,11,12]. Likewise, other programs have used a community approach, focusing on supporting the self-management of chronic illness on behalf of the individual’s social networks [5,13,14,15,16,17]. However, these latter approaches do not provide tie-level measures of the social environments in which self-management may be developed. In addition, they use the terms ‘social support’ and ‘social networks’ interchangeably, conflating two related but unidentical social phenomena. Social support has been defined as ‘support accessible to an individual through social ties to other individuals, groups, and the larger community’ [18]. Instead, social networks have been described as ‘the direct and indirect ties linking a group of individuals over certain definable criteria, such as kinship, friendship, and acquaintances.’ Therefore, social networks provide a structural framework in which support may, or may not, be accessible to the individual [19]. We aim to contribute to the research in this field by providing detailed social tie-level information about the social environment of individuals in situations of chronic pain on one hand, and information about the content of the relationship on the other without prejudging the existence of social support. 

Furthermore, in the study of social support, three differentiated aspects of social ties are distinguished: (1) the existence or number of relationships as a reflection of social integration, (2) the formal structure of the same or social networks, and the functional content, in this case, social support (3) and the influence of the structure of social relationships on functional content in social support [20,21].

Social network analysis (SNA) is a research method that examines the interactions between individuals, groups, and organizations, which has been applied in a variety of research areas. In the context of health, this approach has led to academic literature which has significantly increased in recent decades, prioritizing the role of social structure, including the areas of community and primary health, care and nursing research [22,23,24,25,26], identifying new challenges and providing opportunities for innovative research.

Social support has been studied in several contexts, one of which is informal caregiving [27,28]. Compared to other approaches in social support research, personal network analysis (PNA), also labeled as egocentric network analysis in the literature [29], is based on the ensemble of relationships that surround an individual (ego) across social settings (i.e., family, work colleagues, neighbors,…), as well as the relationships between the person’s contacts (alters) [30,31]. This enables the simultaneous study of micro phenomena (interactions) and meso phenomena (the social networks and institutions that the individual belongs to on a community level) [32]. Previous research has noted the effects of personal networks on individual outcomes based on theoretical frameworks, such as the social capital and the social influence through diffusion and social support, with the latter being one of the most studied areas based both on this approach and on different disciplines [33]. From this perspective, a PNA, constitutes an excellent approach to the study of social support in the context of informal care in chronic illness, since this can be used to measure the structure and composition of personal networks and the functional content of social support. This enables the possibility of differentiating the personal network from the support network (comprising both support providers and non-providers), as well as relating these personal network characteristics, including the structural variables, with other variables of interest. Whereas, in general, social support studies evaluate the quality, or the quantity, of a person’s social ties, studies based on PNA regard these ties as being potentially of interest [34]. This is based on the importance of the relationships between the interacting units [30]. Therefore, the structural variables of these relationships are commonly included in the analysis.

In the study of social support, one dimension of interest is the quality or sense of satisfaction. This dimension reflects the discrepancy among the interactions between real and desired (or necessary) support. The relevance of this distinction is that satisfaction with social support provides a better explanation of the quality of life and health outcomes when compared with the mere provision or number of support providers [35,36]. Regarding the assessment of satisfaction, although it has been used in some instruments intended to evaluate social support [37], there is a gap in the literature regarding the role of the personal networks’ characteristics in the quality or satisfaction with social support. Thus, this study focuses on the quality of social support received from personal networks in the context of chronic pain. This dimension has seldom been researched [36,38] despite being a highly relevant health problem due to its prevalence, complexity, and the consequences for both the individual and the social environment in which it is embedded [39,40]. Our approach provides a rich set of measures at the tie level that allows us to measure with detail personal networks’ structure, composition, and functional content of social support along with its perceived quality. Just having this type of detailed information, it is possible to suggest ways to improve the effective self-management of individuals in chronic pain situations.

## 2. Materials and Methods

This research is part of a larger study aimed at examining social support and quality of life in the context of chronic pain [41]. In a prior publication, the descriptive results of this larger study were presented using a mixed approach [42], showing that different types of personal networks were associated with self-reported quality of life, which scored below the populational mean in all dimensions considered. 

### 2.1. Design

A descriptive, cross-sectional study using personal network analysis. 

### 2.2. Sample Description

The inclusion criteria of participants were people over the age of 18 diagnosed with chronic pain, and receiving care at Marques de Valdecilla University Hospital Pain Unit of Santander (Spain), without mental or cognitive decline and who agreed to participate voluntarily in the study. Convenience sampling was used to select participants: An equal number of men and women (15 in each case) that met the inclusion criteria and were recommended by professionals working at the pain management unit among their patients. All voluntary participants were briefed about the goals and methods of the research and signed an informed consent form (see Section 2.6 below). All of them agreed to be interviewed several times if necessary, either at the hospital or participants’ homes, according to their health status and personal preferences. Nobody requested to quit the research, probably because of the positive assessment of both the attention and feedback about their personal networks they received during the interviews. Fieldwork and data analysis were conducted between July 2014 and July 2015. The personal networks of the 30 cases amounted to a total of 600 personal relationships (20 relationships per ego).

### 2.3. Variables

The data collection included participant variables (ego), their contacts (alter) and details regarding their personal network (see [41,42] for further information regarding the variables):

(a) Sociodemographic data (ego characteristics): sex, age, civil status, level of studies, and work situation.

(b) Variables regarding the composition of the personal network (alter characteristics): Age, sex, type of tie with ego, place of residence, and proximity.

(c) Social support (function): type (emotional, instrumental, informational, and combinations of the same), satisfaction, reciprocity, variation over time, frequency, and channel of transmission.

(d) Variables regarding the structure of the personal network: density, degree centrality (two measures; mean of the alter-alter matrix and at node level), betweenness centrality (two measures; mean of a personal network and at node level), number of components and number of isolates.

### 2.4. Data Collection Instruments

Personal network: EgoNet open-source software (https://sourceforge.net/projects/egonet/), was used to collect and analyze each ego’s personal network data. Additionally, UCInet software [43] was used to calculate degree, and betweenness centrality for each of the 600 alters studied. Sociodemographic, pain variables, and personal network data were collected based on an ad hoc questionnaire designed in accordance with the study objectives.

### 2.5. Data Analysis

Bivariate linear mixed models were used to analyze the satisfaction with the social support received. These were converted into the following numerical values: very unsatisfactory = 0, quite unsatisfactory = 1, satisfactory = 2, quite satisfactory = 3, and very satisfactory = 4. Ego characteristics and structure, composition, and functional content in social support of their personal network were considered as explanatory variables. The ego was included as a random effect for the analysis of the structure and composition of alter variables. Variables with a *p*-value of 0.2 were included in a multivariate logistic regression model to identify which factors were related to satisfactory or very satisfactory support.

Additionally, a multiple correspondence analysis (MCA) combined with classification methods were used in order to establish typologies for the 600 relationships examined in terms of the quality of the social support received from the ego’s point of view. MCA is a descriptive, exploratory technique designed to analyze multi-way contingency tables with cases as rows and categories of variables as columns [44]. Components obtained from the MCA were submitted to a cluster analysis applying Ward’s hierarchical clustering method [45,46]. Results were represented in a tree dendrogram using R-squared distance. Bivariate tests were conducted between each of the explanatory variables and the profiles, using chi-square tests to describe the obtained profiles.

The statistical analysis was performed using SAS v9.4 software (SAS Institute Inc., Cary, NC, USA), and the significance level was set at *p* < 0.05.

### 2.6. Ethical Considerations

The Clinical Research Ethics Committee of Cantabria (Spain) provided ethical approval for this study (internal code 2014.32). All study participants received verbal and written information concerning the study objectives and procedure. Participation was voluntary, and all participants provided their signed informed consent. Furthermore, this study adhered to national and international ethical guidelines (Code of Ethics and Declaration of Helsinki) and fulfilled data confidentiality legislation (Spanish organic law 15/1999 of 13 December on the protection of personal data).

## 3. Results

### 3.1. Sociodemographic Variables

In total, 30 people participated in the study (15 women and 15 men). The mean age of participants was 54.57 years (SD 11.64, range 30–73 years). Their marital status was married or with a partner (*n* = 27); divorced (*n* = 1); and widowed (*n* = 2). Their educational level was primary education (*n* = 16), vocational education (*n* = 8), secondary education (*n* = 4), and higher education (*n =* 2). Their employment status at the time of the interview was: retired (*n* = 10), active (*n* = 9), on sick leave due to pain (*n* = 6), and homemaker (*n* = 5). The mean length of time since the onset of chronic pain was 12.2 years (SD 9.18, range 1–35 years) in men and 16.6 years (SD 12.39, range 1–39) in women.

### 3.2. Bivariate Analysis

The satisfaction with the social support received in the 600 personal relationships studied was distributed as follows: very satisfactory 12.3%, quite satisfactory 22%, satisfactory 26%, quite unsatisfactory 6.5%. The very unsatisfactory category was present in 33.2%, corresponding to relationships where no support was provided (see also [27] for descriptive analysis of other variables related to social support function).

#### 3.2.1. Ego Variables

From the bivariate analysis, no statistically significant relationships were observed (*p*-value > 0.05) between satisfaction with social support received and the following ego variables: Age, gender, level of studies, work situation, civil status, level of pain, pain duration, and the number of cohabitants. However, the results obtained, when comparing the means of age and duration of pain variables for the ego, are notable. The quality of social support received decreased as the ego’s age increased, and as the time with chronic pain increased. 

Age of ego (*p*-value 0.174): for the categorical variable age, as age increased, satisfaction decreased (for every 10 years, satisfaction values decreased by 0.16 units):

- Participants aged between 30 and 51 years; mean satisfaction 2.01 (SD 0.19).
- Participants aged between 52 and 63 years; mean satisfaction 1.72 (SD 0.17).
- Participants aged between 65 and 73 years; mean satisfaction 1.51 (SD 0.18).

Duration of pain (*p*-value 0.096): using the categorical variable model and recoding the duration of pain variable, we observed that, as the duration of pain increased, satisfaction decreased:

- Duration of pain between 1 and 8 years; mean satisfaction 2.04 (SD 0.18).
- Duration of pain between 9 and 19 years; mean satisfaction 1.67 (SD 0.18).
- Duration of pain between 20 and 39 years; mean satisfaction 1.5 (SD 0.18).

#### 3.2.2. Composition Variables

Regarding the composition variables (Table 1), the most satisfactory support was defined by an adult female alter, with whom the ego had a strong tie, who was a close family member (partner, parents, and children) and who lived geographically close.

#### 3.2.3. Structural Variables

Regarding the structural variables of the personal network (Table 2), the quality of the social support received increased as the density of the network increased. In contrast, the quality of support decreased as the number of isolates and components increased. Regarding the node, as the degree centrality of the alter increased (a measure related to the density of the network), satisfaction increased. Likewise, satisfaction increased as the betweenness centrality of the alter increased.

#### 3.2.4. Functional Social Support

Regarding the social support characteristics, of the 600 relationships examined, 401 support-providing relationships were identified (66.83%) and 199 relationships in which support was not provided (33.17%). Thus, the mean number of providers and non-providers was 13 and 7, respectively, for each ego. The functional variables concerning social support (Table 3), reveal that the personal relationships offering greater quality support from the ego’s perspective were characterized by offering various types of support (multiplexity). This was provided face-to-face or combined with daily telephone calls, which could become more frequent over time and occurred in reciprocal support relationships.

### 3.3. Multivariate Logistic Regression Model

From the multivariate logistic regression model, we obtained that the variables which were related to the satisfactory or very satisfactory support were: Age of the ego (the level of satisfaction with the social support received decreased as the age of the ego increased), sex of the alter (women offered more satisfactory support compared to men), tie with the ego (close family members, friends, and neighbors provide more satisfactory support), reciprocal relationships in the support and relationships in which the ego has a very strong tie.

### 3.4. Cluster Analysis

In order to explore the 600 relationships examined in terms of the quality of the social support received a multiple correspondence analysis was performed. Variables included in the analysis were the sex of the alter, tie with the ego, type of support, satisfaction, frequency, transmission channel, reciprocity, and proximity. We obtained 11 factors that accounted for 75% of the total variability. Applying a classification algorithm to these factors, three clusters were obtained, which comprise the totality of the 600 studied relationships. The satisfaction profiles presented the following distribution: Profile 1 (237 relationships), represented by a majority of satisfactory relations with the social support received; Profile 2 (164 relationships), represented by a majority of very and quite satisfactory relationships considering the social support received, and Profile 3 (199 relationships), represented in its totality by very unsatisfactory relationships, and corresponding to the non-providers of social support present in the personal networks studied.

Table 4 displays the distribution of egos among the three profiles according to age. In consonance with the results of the bivariate analysis presented above and which revealed a decrease of satisfaction as the age of the ego increases, the relationships containing cluster 3 and valued as being very unsatisfactory with the social support received corresponded with egos over the age of 65 in almost 50% of cases. 

**Figure 1:**
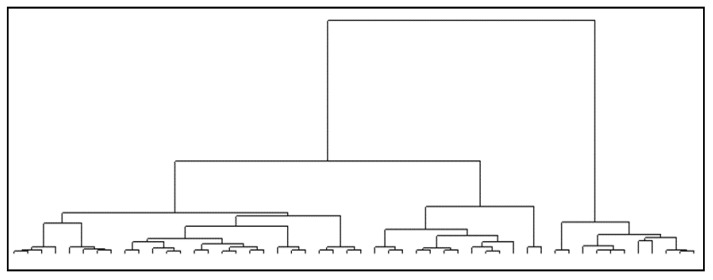
Dendrogram of the hierarchical cluster analysis.

Graphically, these relations are grouped, forming three profiles, as shown in the following dendrogram (Figure 1). The horizontal axis represents individuals that are grouped by horizontal lines at a height that represent the distance between the two linked clusters.

#### 3.4.1. Composition Variables

Table 5 presents the distribution of the personal network composition variables across the three profiles (bivariate tests between the explanatory variables and the profiles). The social support that was deemed more satisfactory was provided by (a) women, (b) close family members (followed by the friends), (c) middle-aged people, (d) those with whom the ego has a strong tie, (e) reciprocal support relationships, and (f) people living geographically close to the ego. Although to a lesser degree, there was a presence of more unsatisfactory relations with non-providers in the case of close adult family members.

For the analysis of the family roles, we have considered: (a) close family members: partner, parents, siblings, and children; (b) family members: Aunts/uncles, grandchildren, cousins, grandparents, nephews/nieces, and brothers/sisters-in-law, and (c) other family members: family roles not included in (a) and (b). The variable ‘proximity’ was examined based on five categories, which were recoded into two categories: A = strong tie: very close, quite close, and close, and (b) weak tie: not very close and not close at all.

#### 3.4.2. Structural Variables

Table 6 presents the multivariate analysis findings considering the importance that structural measures have on the personal network for the quality of the support, in consonance with the results of the bivariate analysis presented above. In this manner, the personal network providing the most highly-valued level of satisfaction combined both a degree centrality and high density with a high betweenness centrality, both of which were above the overall mean. Likewise, the findings reveal a tendency for satisfaction levels to decrease in networks that were more fragmented, with less cohesion or with a greater number of isolates and components.

#### 3.4.3. Functional Social Support

Regarding the specific characteristics of the social support provided (Table 7), the greatest quality was associated with the combination of different types of support, especially emotional and instrumental support. Likewise, the frequency of the provision of support and face-to-face contact or combined with the provision of telephone support represents the most satisfactory support relationships.

**Figure 2:**
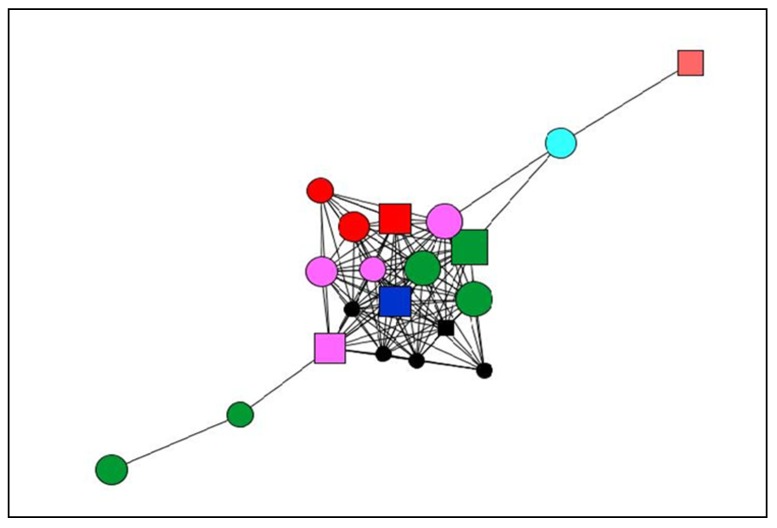
Graph Case 1. Woman (40 years old), living with chronic pain for 14 years.

**Figure 3:**
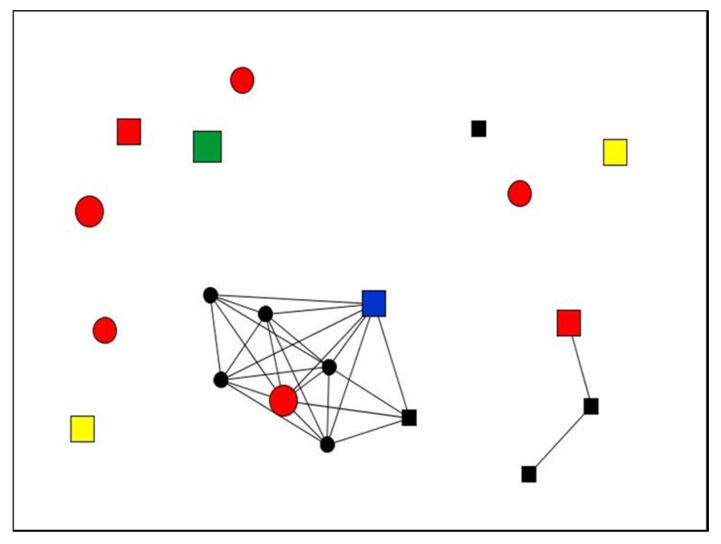
Graph Case 28. Man (66 years old), living with chronic pain for 35 years.

Below, we display the graphs of two study participants with different levels of satisfaction with the social support received. The most satisfactory support network (Figure 2) corresponds to a woman aged 40 years who had chronic pain for 14 years, whereas the graph that illustrates the least satisfactory support network (Figure 3) is that of a man aged 66 years who had experienced chronic pain for 35 years. Table 8 shows the legend of graphs, and Table 9 displays the quantitative results of the differences in these three personal network dimensions examined, as well as the quality of social support.

In addition to the differences of age and time since onset of pain in each of the two cases presented, the comparison of these two networks reveals differences affecting the structure, composition, and content of the social support as well as the satisfaction with the social support received by each of the two participants.

## 4. Discussion

This study aimed to examine the structure, composition, and functional content of social support in the context of personal networks, in relation to satisfaction from the ego perspective. It is important to consider these conditions in order to assess social support as a relational element that is contained and transmitted within social relations [20]. 

### 4.1. Composition Variables

The most highly-valued social support was provided by middle-aged female alters, with whom the ego maintained a close relationship and who lived geographically close to the ego. These characteristics highlight the importance of the age of the alter on the ability to offer support (children and older people as non-providers) or the geographical proximity of the alter, which is considered key, especially for the provision of instrumental or tangible support [47,48,49]. Regarding the type of tie with the ego, our results add to the existing literature, which underlines the significant role of the family and friends in the provision of social support [50]. Nonetheless, it is necessary to consider that, although family roles represent 76.8% of the most satisfactory relationships, they also represent 55.8% of the total number of relationships in the profile identified as the most unsatisfied with the social support received. Therefore, when considering social support in the framework of social relationships, it should be appreciated that not all aspects of so-called close relationships are positive [20,51]. Negative interactions, together with social loss and loneliness, constitute adverse aspects of social interactions [52], which can be detrimental to a person’s health by influencing the sense of wellbeing, life stress, less supportive networks, and psychological distress [53,54,55]. Likewise, specifically in cases of chronic pain, it is also necessary to consider the impact of pain and the resultant effects on a person’s social and family relationships [39,40,56], including effects on their partners [57,58,59,60]. Therefore, these findings suggest the need for contextual and longitudinal assessments of support in long-term conditions, as these factors affect both the receiver, as well as the provider of support and the relationship dynamics. 

### 4.2. Structural Variables

Our results have shown that quality in the provision of social support is related to certain levels of density as well as with a relatively high betweenness centrality. Nonetheless, none of these explain separately the maximum level of satisfaction. Therefore, the results suggest that the ideal support network should strive for a certain balance between a dense center and a periphery that may act as a bridge with other more diverse relationships.

From a sociological point of view, social structures affect and are affected by human behavior [61]. In the field of health, several studies have underlined the role that the network structure plays in different health outcomes, such as the effect of the same on health-related behaviors [62,63,64] in the transmission of sexually transmitted diseases [65,66] in mental health [67] or the relation between network structure and health status [68]. Likewise, some studies have found certain benefits derived from having a network with different social domains and with a diverse typology of alters, as these can act as facilitators of other resources offering different types of support [69,70]. Therefore, an alter with a high betweenness, as shown by our results, represents a quality support resource for ego, which can benefit from these indirect relationships. Conversely, some studies have found that density is not automatically related to social support [71,72] and that networks with low density are more adaptive and offer greater support according to determined contexts [73]. We contend that this idea of *balance* among density and intermediation in the structure of the personal network is important when evaluating social support. For the case of chronic pain, a dense network may guarantee the availability of emotional support in which strong ties mainly are represented by kin and close relationships. However, the same relationships over time may lead to a redundancy of the already-known support resources, and alters with a high betweenness might enable access to new support resources (information, contacts, etc.) of great value for ego. Although certain levels of density are necessary for achieving a feeling of safety, something necessary in a healthy personal context, the effectiveness of the network in relation to social support is based on ties that allow access to diverse resources with alternative ways of thinking and acting [74,75], and that the person may use as ‘social capital’ assets [76]. Therefore, in certain contexts, density and intermediation offer different advantages and may, in fact, be complementary [77].

### 4.3. Functional Social Support

Concerning the types of support provided, emotional support was most highly-valued, followed by the combination of emotional and instrumental support, reinforcing the importance of a multiplexity or diversity of resources [34]. Thus, in the context of chronic pain, emotional support may guarantee a feeling of accompaniment, understanding, and empathy for the other person’s situation, as well as constituting a coping resource, enhancing the ability to adapt to the situation and acting as a facilitator of self-management. Furthermore, in the case of chronic pain, having the instrumental support of others is necessary because of the impairments associated with the performance of basic activities of daily living such as, for example, mobilization, hygiene, or personal grooming. Our findings support previous research [78,79,80] showing that relationships characterized by reciprocal support are those that are most highly-valued, highlighting the importance of this characteristic in relationships involving health aspects. In addition, increasing and frequent face-to-face support is a characteristic of the most highly-valued support relationships. It is important to note that these findings may be related to caregiver burden as a consequence of caring for people with chronic illnesses in the long term [81,82,83].

According to previous studies [84], and highlighting the importance of the dynamic nature of personal relationships throughout the life cycle, our findings reveal that satisfaction with the social support received decreases as the age of the ego increases and as the time since the onset of pain increases. This aspect is highly relevant in chronic illness and, more specifically, in the case of chronic pain, mainly because of: (a) the increased prevalence of this disorder in aging populations [39,56], (b) the changes in personal networks at the ego-alter level, i.e., the alters that are lost and are added to the personal network over time, and (c) changes in the characteristics of the relationships as a consequence of life events (e.g., marriage, divorce, chronic illness, retirement, etc.) that may affect the content in social support [85,86,87]. 

Personal contexts, namely the characteristics of relationships and personal networks, are key elements that help us understand the complexity of satisfaction with social support for self-management in chronic illnesses. 

Future research with a longitudinal design is recommended, focused on the study of variations in social support over time in individuals with chronic pain. Lastly, comparative studies involving personal network research may reveal possible differences in structure, composition, and content in the social support of personal networks according to the age of the ego, as well as providing further information on the support networks of older people.

## 5. Conclusions

The satisfaction with the informal care received by people with chronic pain, via their personal network resources, is different according to personal characteristics, such as age, pain, the amount of time since the onset of chronic pain, as well as characteristics related to their personal network. Person-centered care implies considering the different social and relational contexts in which people live their lives. Support for self-management in situations of chronic illness includes the support provided by primary care nursing professionals and health organizations via different strategies, as well as the support from the social and personal environment surrounding the person and, therefore, both can be considered as being complementary. From the point of view of the support provided by the personal network, we have shown that a balance between degree centrality and betweenness (an indicator of the existence of various social circles connected through the ego) is needed in order to achieve higher satisfaction with the support received. This finding may help to enhance the self-management capabilities of this type of patient by introducing small adjustments to their personal network structures. In this vein, cases in which insufficient or inappropriate social support are detected in an individual’s personal environment could benefit from the implementation of strategies, based on specially designed network interventions, with the aim of guaranteeing the continuity and appropriateness of care and support over time.

## Figures and Tables

**Table 1:** Satisfaction and composition variables of the personal network.

Variable	Category	Mean (SD)
Age of the alter *	≤30 years	1.36 (0.17)
	31–50 years	1.95 (0.13)
	51–60 years	1.67 (0.16)
	>60 years	1.69 (0.14)
Sex of the alter *	Women	1.91 (0.12)
	Men	1.56 (0.12)
Tie with the ego *	Partner	3.22 (0.26)
	Parents	3.03 (0.33)
	Brothers	1.75 (0.18)
	Children	2.77 (0.19)
	Other family members	1.32 (0.13)
	Friends	1.61 (0.14)
	Other ties	1.41 (0.18)
Strength of the tie *	Very close	2.83 (0.13)
	Quite close	2.19 (0.12)
	Close	1.50 (0.12)
	Not very close	0.74 (0.13)
	Not at all close	0.21 (0.20)
**Place of residence of the alter compared to the ego**	Same locality	1.89 (0.13)
	Same province	1.66 (0.13)
	Other province/country	1.53 (0.18)

* *p*-value < 0.05.

**Table 2:** Satisfaction and structure variables of the personal network and alters.

Variable	Range/Category	Mean (SD)
Density *	<40%	1.57 (0.15)
	41–60%	1.64 (0.16)
	>60%	2.28 (0.22)
Degree centrality (alter) *	0–5	1.35 (0.14)
	6–11	1.60 (0.13)
	12–19	2.35 (0.15)
Betweeness Centrality (alter) *	0	1.44 (0.13)
	0.1–1.2	1.69 (0.15)
	1.2–114.3	2.12 (0.14)
Components	1	2 (0.16)
	2	1.54 (0.20)
	3–11	1.58 (0.18)
Isolates	0	1.89 (0.14)
	1	1.63 (0.23)
	2–9	1.46 (0.22)

* *p*-value < 0.05.

**Table 3:** Satisfaction and social support variables.

Variable	Category	Mean (SE)
Social support variables in provider relationships (*n* = 401)		
Type *	Emotional	2.37 (0.07)
	Emotional and instrumental	3.18 (0.10)
	Emotional, instrumental and informational	3.62 (0.18)
	Other types of support	2.55 (0.14)
Frequency *	Daily	3.26 (0.09)
	Weekly	2.57 (0.08)
	Biweekly	2.42 (0.12)
	Monthly	1.94 (0.11)
	>2 months	1.83 (0.15)
Transmission channel *	Face-to-face	2.70 (0.09)
	By telephone	2.22 (0.13)
	Face-to-face and by telephone	2.74 (0.10)
	Internet/Telephone + internet	2.16 (0.28)
Variation of support *	Increases	3.22 (0.18)
	No variation	1.47 (0.12)
	Decreases	1.73 (0.19)
Reciprocity *	Yes	1.97 (0.10)
	No	0.99 (0.14)

SE = Standard Error.* *p*-value < 0.05.

**Table 4:** Age of ego and relationships according to the three profiles (%).

Category	Profile 1	Profile 2	Profile 3
30–49 years	38.4	31.1	19.1
50–64 years	35.9	37.2	37.2
65 and over	25.7	31.7	43.7
Total	100	100	100

**Table 5:** Composition variables of the personal network according to the three profiles (%).

Variable	Category	Profile 1	Profile 2	Profile 3
Age of the alter *	<20	2.1	7.3	15.1
	20–39	21.5	24.4	19.6
	40–59	45.6	45.2	32.7
	>60	30.8	23.1	32.6
Sex of the alter *	Male	45.1	41.5	57.8
	Female	54.9	58.5	42.2
Relationship with ego *	Close family members	17.3	59.8	14.1
	Family members	24.9	7.9	24.1
	Other family member	4.2	9.1	17.6
	Friends	35.9	17.1	32.2
	Neighbors	8.4	3	6.5
	Work and professional colleagues	9.3	3	5.5
Strength of the tie *	Strong tie	77.2	100	49.2
	Weak tie	22.8	0	50.8
Place of residence of the alter compared to the ego *	Same location	35	58	43.2
	Same province	43	32.9	40.2
	Other province	17.4	8.5	12.6
	Other province/country	4.6	0.6	4

* *p*-value < 0.05.

**Table 6:** Structural variables of the personal network. Profile and global means.

Variable	Profile 1	Profile 2	Profile 3	Global Mean
Density	0.44	0.53	0.41	0.45
Degree Centrality ^(a)^	7.08	12.44	7.25	8.60
Betweeness Centrality ^(a)^	2.41	8.53	2.10	3.98
Components	2.41	2.11	3.03	2.53
Isolates	0.91	0.74	1.62	1.10

^(a)^ Alters’ centrality measures

**Table 7:** Social support variables in the three profiles (%).

Variable	Category	Profile 1	Profile 2	Profile 3
Type *	Emotional	83.5	34.8	0
	Instrumental	5.1	2.4	0
	Informative	2.1	0	0
	All three types of support	0.4	12.8	0
	Emotional and instrumental	3.4	46.3	0
	Emotional and informative	3.4	3.7	0
	Instrumental and informative	0.4	0	0
	Professional	1.7	0	0
	None	0	0	100
Frequency *	Daily	7.6	63.4	0.5
	Weekly	39.2	31.7	0.5
	Biweekly	17.3	3.7	0
	Monthly	23.6	0.6	0
	Every 2 or 3 months	6.3	0.6	0
	Every 3 months or more	5.9	0	99
Channel of transmission *	Face-to-face	43.5	54.3	0
	By telephone	24.1	3.7	0
	Internet	3	0.6	0
	Face-to-face and by telephone	27.8	41.5	0
	Telephone and internet	1.7	0	0
	No support	0	0	100
Variation *	Has not varied	70.5	61	92
	More support	13.1	31.1	0
	Less support	16.5	7.9	8
Reciprocity *	Yes	78.9	93.3	59.3

* *p*-value < 0.05.

**Table 8:** Legend of graphs.

Node Shape: Sex	Node Size: Satisfaction	Node Colour: Type of Social Support
Circle: Women Square: Men	Large: More satisfaction Small: Less satisfaction	Red: Emotional Dark blue: Instrumental Pink: Emotional and instrumental Yellow: Informational Green: All types Black: Non-providers Light blue: Emotional and informational Orange: Professional

**Table 9:** Descriptive analysis of social support at the ego level.

		Case 01	Case 28
Satisfaction (%)	Very satisfactory	20	0
	Quite satisfactory	35	15
	Satisfactory	20	40
	Quite unsatisfactory	0	0
	Very unsatisfactory	25	45
Structure	Density	0.605	0.137
	Degree Centrality (mean)	11.5	2.6
	Betweenness Centrality (mean)	5.7	0.25
	Components	1	11
	Isolates	0	9
Composition (%)	Sex of the alters Women (vs. men)	70	50
	Strength of the tie Strong tie (vs. weak)	80	90
	Place of residence of alters Same locality as ego	80	15
	Reciprocity Yes	45	90
Social support (%)	Type of support All three types Emotional and instrumental Non-providers	25 20 25	5 0 45
	Variation of support Increases	55	15
	Frequency of support Daily 2 or 3 times per week	25 10	5 0
	Transmission channel Face-to-face Face-to-face and telephone	65 10	45 10
